# 1568. Evaluating the Appropriateness of Antiretroviral Therapy in the Critical Care Setting

**DOI:** 10.1093/ofid/ofad500.1403

**Published:** 2023-11-27

**Authors:** Toobah Wali, Barbara Kamel, Thien-Ly Doan

**Affiliations:** Long Island Jewish Medical Center, New Hyde Park, New York; Long Island Jewish Medical Center, New Hyde Park, New York; Long Island Jewish Medical Center, New Hyde Park, New York

## Abstract

**Background:**

The care of patients with HIV in the intensive care unit (ICU) can be complex as they may develop acute kidney injury and therefore necessitating continuous renal replacement therapy, need feeding tubes, and are prescribed new medications. Issues regarding drug-drug interactions, fluctuating renal & hepatic function, adverse effects, oral administration issues including crushing of tablets are challenges that may arise. The purpose of this study was to evaluate the appropriateness of antiretroviral (ARV) therapy in the ICU.

Background - HIV and ICUs

**Figure:**
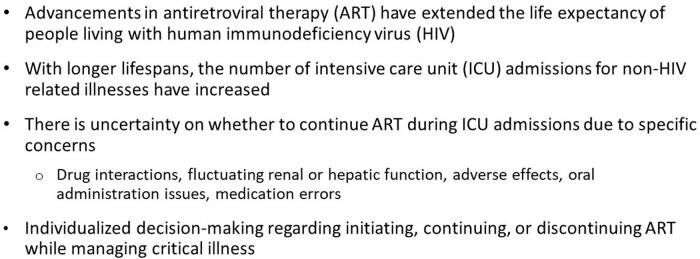

In 2018, almost half of Americans living with HIV were aged 50 and older. Co-morbidities associated with ICU admissions include cardiovascular, renal, hepatic, and pulmonary disease. Medication errors are defined as: incorrect/incomplete regimen, incorrect dose, incorrect renal adjustment, incorrect administration, and major drug-drug interaction.

**Methods:**

This was a retrospective, observational, quality improvement project that reviewed charts from January 2018 to August 2022. Adult patients that were admitted to the the ICU at Long Island Jewish Medical Center and receiving ART for HIV were included. Patients receiving ARV for indications other than HIV were excluded. Using the medical record system, data was collected: demographics, renal and hepatic dysfunction, ARV ordered (e.g., tablet formulation, combination/single tablet), completeness of treatment regimen, drug-drug interactions, feeding placement, and concomitant medications. Descriptive statistics were utilized.

**Figure:**
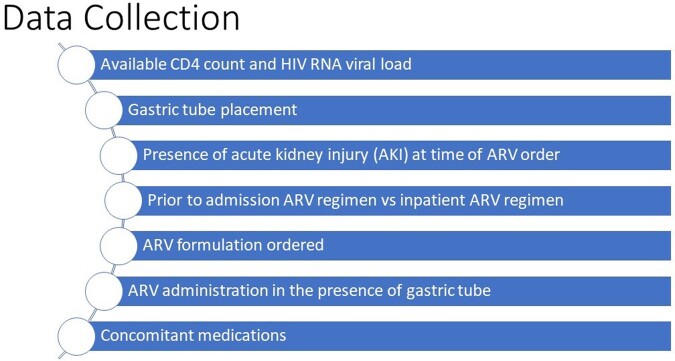

**Results:**

Of the 68 patients screened, 43 were included in the study. The mean age was 57 ± 13 years, 60.5% males, and majority of patients were admitted to the medical ICU (55.8%). The median CD4 count was 250 cells/μL and 37.2% of the patients had undetectable viral loads. A total of 18 patients (41.9%) required a gastric tube during their ICU stay. ID was consulted in 41.9% of the cases. Inappropriate ART was found in 46.5% of patients with drug-drug interactions, inappropriate tablet crushing, and renal dose adjustment needed being the most prevalent reasons for inappropriateness. 27 patients (62.8%) had opportunities for interventions. which included failure to re-initiate ARV sooner (n = 8), inappropriate ARV crushing (n = 8), ARV use contraindicated at time prescribed (n = 6), ARV not reconciled once stable (n = 5), and drug-drug interactions (n = 4). An intervention could have been made 5 days sooner on average.

**Figure:**
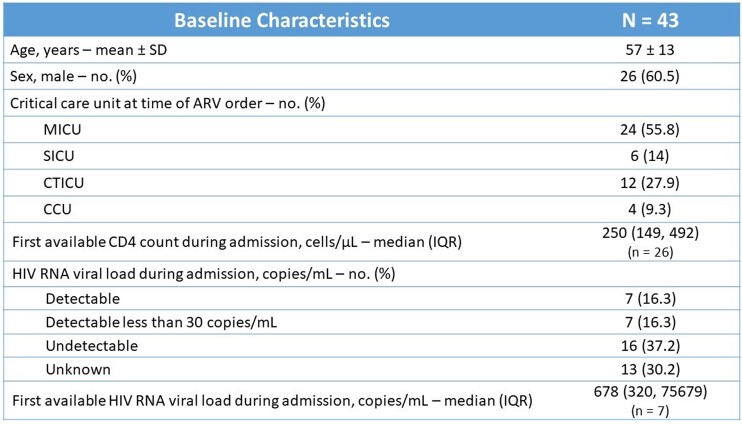

**Figure:**
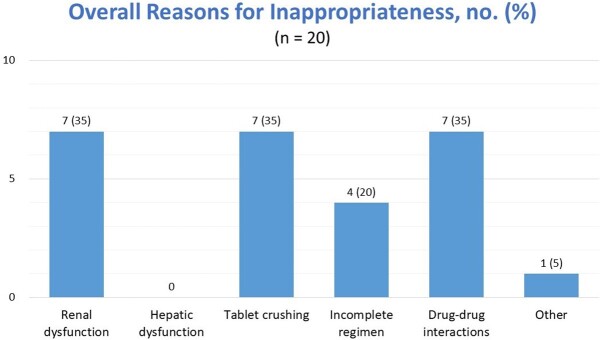

20 patients identified with an inappropriate ART regimen and patients could have had more than one reason for inappropriateness so total 26 identified reasons for inappropriate ART Most reasons for inappropriateness were related to patient’s renal function, ART administration, and drug-drug interactions followed by incomplete regimen. The other reason for inappropriateness was that patient was not re-initiated on ART during ICU stay when it was appropriate to do so.

**Figure:**
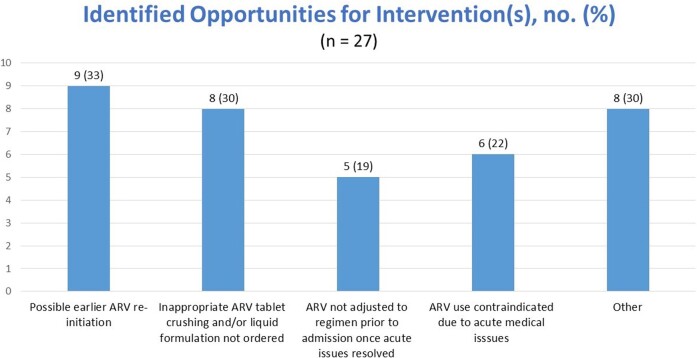

27 patients identified with one or more areas for improvement– these were interventions that could have been made. Most areas for improvement included earlier ARV re-initiation either to the same regimen from home PTA or a more appropriate regimen (such as a kidney or liver-sparring regimen) Other areas identified included inappropriate formulation/administration of ART, contraindications such as AKI were present at the time ART was ordered, addressing drug-drug interactions, Other = address drug-drug interactions (4), earlier initiation of new ARV regimen (patient was tx-naïve) (1), earlier initiation of adjusted ARV regimen (1), incomplete regimen (1)

**Conclusion:**

The study demonstrates that there are opportunities for improvement in the care of HIV in the ICU setting. Daily assessments of ART for re-initiation, dose adjustment, appropriate administration is crucial.

**Figure:**
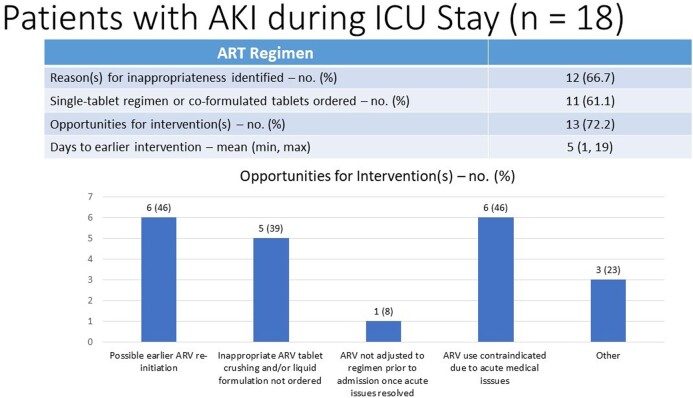

**Figure:**
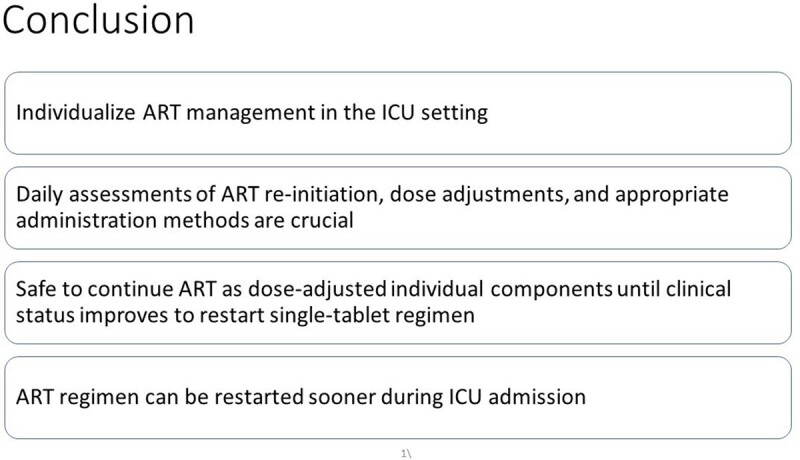

**Disclosures:**

**All Authors**: No reported disclosures

